# Factors associated with adoption of beneficial newborn care practices in rural Eastern Uganda: a cross-sectional study

**DOI:** 10.1186/s12884-016-0874-3

**Published:** 2016-04-21

**Authors:** Michael O. Owor, Joseph K. B. Matovu, Daniel Murokora, Rhoda K. Wanyenze, Peter Waiswa

**Affiliations:** Makerere University School of Public Health-CDC Fellowship Program, P.O. Box 7072, Kampala, Uganda; Department of Health Policy Planning and Management, Makerere University School of Public Health, P.O.Box 7072, Kampala, Uganda; Baylor Uganda Children’s Foundation, P.O. Box 72052, Kampala, Uganda

**Keywords:** Beneficial newborn care practices, Maternal characteristics, Socio-economic status

## Abstract

**Background:**

Beneficial newborn care practices can improve newborn survival. However, little is known about the factors that affect adoption of these practices.

**Methods:**

Cross-sectional study conducted among 1,616 mothers who had delivered in the past year in two health sub-districts (Luuka and Buyende) in Eastern Uganda. Data collection took place between November and December 2011. Data were collected on socio-demographic and economic characteristics, antenatal care visits, skilled delivery attendance, parity, distance to health facility and early newborn care knowledge and practices. Descriptive statistics were computed to determine the proportion of mothers who adopted beneficial newborn care practices (optimal thermal care; good feeding practices; weighing and immunizing the baby immediately after birth; and good cord care) during the neonatal period. We conducted multivariable logistic regression to assess the covariates of adoption of all beneficial newborn care practices. Analysis was done using STATA statistical software, version 12.1.

**Results:**

Of the 1,616 mothers enrolled, 622 (38.5 %) were aged 25-34; 1,472 (91.1 %) were married; 1,096 (67.8 %) had primary education; while 1,357 (84 %) were laborers or peasants. Utilization of all beneficial newborn care practices was 11.7 %; lower in Luuka (9.4 %, *n* = 797) than in Buyende health sub-district (13.9 %, *n* = 819; *p* = 0.005). Good cord care (83.6 % in Luuka; 95 % in Buyende) and immunization of newborn (80.7 % in Luuka; 82.5 % in Buyende) were the most prevalent newborn care practices reported by mothers. At the multivariable analysis, number of ANC visits (3-4 vs. 1-2: Adjusted (Adj.) Odds Ratio (OR) = 1.69, 95 % CI = 1.13, 2.52), skilled delivery (Adj. OR = 2.66, 95 % CI = 1.92, 3.69), socio-economic status (middle vs. low: Adj. OR = 1.57, 95 % CI = 1.09, 2.26) were positively associated with adoption of all beneficial newborn care practices among mothers.

**Conclusion:**

Adoption of all beneficial newborn care practices was low, although associated with higher ANC visits; middle-level socio-economic status and skilled delivery attendance. These findings suggest a need for interventions to improve quality ANC and skilled delivery attendance as well as targeting of women with low and high socio-economic status with newborn care health educational messages, improved work conditions for breastfeeding, and supportive policies at national level for uptake of newborn care practices.

## Background

In 2005, Lawn et al. estimated that about 4 million neonatal deaths occur every year in the first 4 weeks of life, contributing 38 % of the estimated 10.5 million deaths that occur among children under-5 years of age worldwide. The highest numbers of neonatal deaths occur in sub-Saharan Africa. Few countries (with some exceptions) have made progress in reducing such deaths in the past 10–15 years [1]. In 2013, neonatal dates still accounted for 41.6 % of under-5 mortality. The rising numbers of births especially in sub-Saharan Africa have lead to more 1.4 million more child deaths [2]. The precursors of these neonatal deaths have partly been traced back to lack of practice of beneficial newborn care practices and attributable to largely infections, intrapartum birth conditions, and preterm births [3–5]. According to WHO, Save The Children and UNICEF [6–8], beneficial newborn care practices are described as interventions such as immunization of baby (with birth dose of oral polio and Hepatitis B vaccines) after birth [9], assessment for birth weight, gestational age, congenital defects and signs of newborn illness [6], good cord care practices such as use of antiseptics to dry the umbilical care [10, 11], skin to skin care and maintaining warmth [11–13], as well as early and exclusive breastfeeding [14–16]. Studies have shown that beneficial newborn care practices at population level can save many newborn lives [11, 17, 18]. In addition, these beneficial newborn care interventions are simple, cost effective, and acceptable: a single skilled birth attendant caring can effectively provide many of them for the mother and the newborn and sometimes by the mother herself if taught [1, 16, 19–22].

In Uganda, studies have demonstrated low levels of newborn care knowledge among certain cadres of health workers-particularly midwives: a gap in the promotion of beneficial newborn care practices among newborn mothers and caregivers [23]. In addition, disparities in equitable access to health care are likely to hinder promotion of beneficial newborn care practices among mothers and caregivers since 52 % of deliveries do not take place in health facilities [24, 25]. This creates a gap in delivery of these interventions at health system level in addition to the existing social inequity to healthcare access among the rural poor [3, 16, 26, 27]. The government of Uganda, like other high burden states, has undertaken several steps including policy action to prioritize newborn health, improving midwife coverage and health system strengthening to help improve equitable access to healthcare [28–30]. Additionally, she has defined a minimum package of health services for all levels of healthcare, abolished user fees, and ensured the existence of a functional health facility within reach (within 5 km) of the majority of households [6]. Furthermore, the Ugandan Ministry of Health has strengthened community support systems through use of community health workers such as village health teams (VHTs) to create demand for services and improve adoption of beneficial newborn care practices among caregivers and newborn mothers, especially in the early newborn period [31, 32].

Despite these efforts, access to newborn health care services remains inadequate in most parts of the country, and adoption of beneficial newborn care practices remains largely sub-optimal [3, 33]. Few studies, if any, have explored the factors associated with adoption of beneficial newborn care practices among mothers [3, 23], thereby presenting a missed opportunity for improving neonatal care in Uganda. We assessed prevailing beneficial newborn care practices among mothers resident in Buyende and Luuka health sub-district in eastern Uganda; and assessed the factors associated with adoption of these practices in the two health sub-districts. Our study findings will help to inform policy on implementation and rollout of the newborn care aspects of the child survival strategy in the light of high neonatal mortality in Uganda and to track progress of newborn care interventions [24, 34–37].

## Methods

### Study design, setting, participants

This was a cross-sectional household baseline study conducted among 1,616 mothers in two rural health sub-districts of Buyende and Luuka in Eastern Uganda. Both Buyende and Luuka health sub-districts are within Buyende and Luuka districts respectively, which are part of Busoga region contributing 10 % of the population of Uganda. Over 80 % of the population are peasants and live on less than US$1 a day. The crude birth rate in both districts (Buyende and Luuka) averages that of the country at 42 live births per 1,000 populations [3, 38]. Household interviews were conducted between November and December 2011. Mothers, who provided written informed consent, had given birth in the last 1 year, and had live babies, were exhaustively recruited. We excluded those who had stillbirths or whose babies died prior to interview to minimize the social consequences associated with asking mothers about babies that died immediately after birth.

### Sampling and sample size determination

Buyende and Luuka are two of the control health sub-districts in Eastern Uganda where The Maternal Newborn Study (MANEST) was implemented. MANEST was a quasi-experimental 30 months study that started July 2011 and ended December 2013. The goal of the study was to learn how to integrate and scale-up interventions aimed at increasing access to institutional deliveries and care of complications through vouchers, and improving newborn care and uptake of Prevention of Mother to Child transmission of HIV (PMTCT) through home visits by community health workers, within the existing health system in Uganda. As part of the baseline assessment, to inform the final design of the intervention, data were collected among 1616 mothers in both health sub-districts who met the eligibility criteria i.e. had given birth in the last 1 year and had provided written informed consent and live babies.

### Data collection procedures

Data were collected using paper-based questionnaires by trained research assistants. The quality assurance of data was ensured through daily assessment via questionnaires filled-in by a supervisor; in cases of error or incompleteness of data, corrective measures were implemented immediately i.e. mothers were re-visited to ascertain correctness of the data, except for data they could not recall.

### Measures

- Outcome measures

The primary outcome was the proportion of women who reported that they adopted beneficial newborn care (NBC) practices. Beneficial NBC practices were grouped into five categories: (i) Optimal thermal care defined as: newborn after birth, was first dried, put skin-to-skin on mothers chest, wrapped in clean dry clothing and delayed bathe (after 24 h or more), (ii) Good cord care defined as: type of instrument used to cut the cord (such as a brand new razor blade, surgical blade or sterilized pair of scissors), type of material used to tie the cord (clean thread), and no medicinal substance (local or not local) put on the cord), (iii) Good feeding practices defined as: initiating breastfeeding within the first 1 h after birth and exclusively breastfeeding in the first month of life, (iv) Weighing the baby immediately after birth, and (v) immunization (if the baby was given oral polio vaccine (OPV) and/or BCG after birth). These NBC practices were further combined into an index of all beneficial NBC practices, which was dichotomized as (“Yes = 1”, if the mother practiced all the beneficial newborn care practices and “No = 0”, if the mother practiced none or just a few).

b) Explanatory measures

Age distribution was checked for normality and found to be skewed (to the right). We then categorized age as follows: ≤24 (less than or equal to twenty-four years), 25–34 (Twenty-five to thirty-four years) and 35+ (Thirty-five years and above). Parity (number of pregnancies carried beyond 28 weeks) of mother was grouped into 1, 2-4 and 5+, while Trimester at first ANC was categorized according to weeks of gestation when the mother had her first ANC visit as follows: trimester 1 < 13 weeks, trimester 2 = 14–26 weeks and trimester 3 = 27–40 weeks. Number of ANC visits was categorized into 1-2, 3-4, and 5+. Distance to health facility where mother delivered was categorized into (< or =5 km, >5 km and not known). The other variables i.e. Marital Status, education level, occupation, husband’s education, skilled delivery (delivery by midwife, doctor clinical officer or nurse at a facility), delivery mode, ANC visit, were left intact.

To generate household socio-economic status (SES), we considered the following variables: floor material, roof material, wall material, fuel used for cooking, source of light and other household possessions (i.e. radio, type of bed, table refrigerator, television set, sound cassette player, and telephone), agricultural land, and farm animals (chicken, goats, cows, pigs, sheep). These variables were screened for relevance and reliability using Cronbach’s alpha (which was found to be 0.628) and acceptable [39]. The final list of variables included agricultural land, type of floor material, type of roof material, wall material, fuel used for cooking, and source of light. We performed Principal Component Analysis (PCA), scored the first principal component, and used it to generate an asset index. The asset index was then used to group all households into wealth quartiles; i.e., <25 % = Lowest, 25–50 % = Low, 50–75 % = Middle and >75 % = High socio-economic status) [40]. We merged the ‘lowest’ and ‘low’ quartiles into “low” because lowest had very few values while “middle” and “high” were left intact. This resulted in three socio-economic status levels, namely: low, middle and high, as presented in the Tables.

### Statistical analyses

We computed descriptive statistics to determine the proportion of mothers who adopted beneficial newborn care practices separately for each health sub-district and conducted bivariate analyses using Pearson chi2 test to assess the association between adoption of beneficial newborn care practice and mothers socio-demographic and other characteristics. Before inclusion in the model, we assessed for collinearity of the explanatory variables and there were none. All variables with a *p*-value less than 0.1 (*p* < 0.1) at the bivariate analysis were included in the multivariable analysis. We then conducted multivariable logistic regression analyses using the likelihood ratio test to assess the covariates of a mother adopting all beneficial newborn care practices after adjusting for number of ANC visits, skilled delivery, husband’s education status, education; occupation, delivery mode, trimester at first ANC visit, socio-economic status, and health sub-district. Missing values accounting for 2 % in the final model were excluded from the analysis. A *p*-value less than 0.05 (p < 0.05) was considered significant at the multivariable analysis.

While we intended to run separate multivariable regression models for each health sub-district, we were not able to do this due to the limited number of women reporting adoption of all the beneficial newborn care practices in each health sub-district. In order to account for the differences in adoption of beneficial newborn care practices between the two health sub-districts, we controlled for health sub-district of residence in the adjusted analysis. The Hosmer and Lemeshow’s goodness-of-fit test was used to assess how the final multivariable model fit the data and was found to be 0.896, 8 d.f, *p* = 0.35, which was satisfactory. We estimate that this study had a post-hoc statistical power of 81 % to detect an odds ratio of 0.64 as significant at an alpha-level of 0.05 when comparing adoption of all beneficial newborn care practices between the two health sub-districts. Data were analyzed using STATA version 12.1.

### Ethical considerations

Makerere University School of Public Health Institutional Review Board approved the study. Written Informed consent was sought from study participants after reading to them and adequately explaining to them the aim of the study. Participants were informed of their right to withdraw from the study at any stage of the interview.

## Results

A total of 1,616 mothers were enrolled into this study. Of these, 797 (49.3) were enrolled from Luuka while 819 (50.7) were enrolled from Buyende health sub-district (Table 1). Of the 1,616 mothers enrolled, 622 (38.4 %) were aged 25–34; 1,096 (67.8 %) had primary education, 1,472 (91.1 %) were married; while 1,357 (84 %) were laborers or peasants. Forty four per cent (726) of the mothers were of parity 5+; 1,583 (98 %) reported antenatal care attendance; 979 (61.8 %) attended ANC for 3-4 times while 889 (56.2 %) had their first ANC visit in trimester 2. Skilled delivery was reported among 1,183 (73.2 %) of mothers while 1,569 (97.1 %) of mothers had a normal delivery. Distance to place of delivery was not known among majority of mothers 1,154 (71.4 %).

**Table 1:** Population characteristics stratified by health sub-district of residence

	Health sub-district		
Characteristics	Luuka *N* = 797 (%)	Buyende *N* = 819 (%)	Total *N* = 1,616 (%)
Age group			
≤ 24	296 (37.1)	271 (33.1)	567 (35.1)
25–34	309 (38.8)	313 (38.2)	622 (38.5)
35+	157 (19.7)	166 (20.3)	323 (20.0)
Missing Values	35 (4.4)	69 (8.4)	104 (6.4)
Marital Status			
Not married	77 (9.7)	67 (8.2)	144 (8.9)
Married	720 (90.3)	752 (91.8)	1,472 (91.1)
Education			
No education	64 (8.0)	130 (15.9)	194 (12.0)
Primary	534 (67.0)	562 (68.6)	1,096 (67.8)
Secondary or higher	199 (25.0)	127 (15.5)	326 (20.2)
Occupation			
Salaried or business	61 (7.7)	52 (6.3)	113 (7.0)
Laborer or peasant	652 (81.8)	705 (86.1)	1,357 (84.0)
Housewife or other	84 (10.5)	62 (7.6)	146 (9.0)
Husband’s Education Status			
No education	135 (16.9)	157 (19.2)	292 (18.1)
Primary	358 (44.9)	424 (51.8)	782 (48.4)
Secondary or higher	304 (38.2)	238 (29.0)	542 (33.5)
Attended ANC			
No	14 (1.8)	17 (2.1)	33 (1.9)
Yes	782 (98.1)	801 (97.8)	1,583 (98.0)
Missing Values	1(0.1)	1(0.1)	2(0.1)
Number of ANC Visits^a^			
1-2	139 (17.8)	164 (20.5)	303 (19.1)
3-4	474 (60.6)	505 (63.0)	797 (61.8)
5+	169 (21.6)	132 (16.5)	301 (19.1)
Trimester at first ANC Visit^a^			
Trimester 1	292 (37.3)	272 (34.0)	564 (35.6)
Trimester 2	438 (56.0)	451 (56.3)	889 (56.2)
Trimester 3	52 (6.7)	78 (9.7)	130 (8.2)
Parity			
1	125 (15.7)	107 (13.1)	232 (14.4)
2–4	315 (39.5)	343 (41.9)	658 (40.7)
5+	357 (44.8)	369 (45.0)	726 (44.9)
Skilled Delivery			
No	192 (24.1)	241 (29.4)	433 (26.8)
Yes	605 (75.9)	578 (70.6)	1,183 (73.2)
Delivery Mode			
Assisted	23 (2.9)	24 (2.9)	47 (2.9)
Normal delivery	774 (97.1)	795 (97.1)	1,569 (97.1)
Distance to Health Facility where Mother Delivered from			
≤ 5 km	113 (14.2)	117 (14.3)	230 (14.2)
> 5 km	101 (12.7)	131 (16.0)	232 (14.4)
Distance not known	583 (73.1)	571 (69.7)	1,154 (71.4)
Socio-economic Status			
Low	180 (22.6)	300 (36.6)	480 (29.7)
Middle	346 (43.4)	345 (42.1)	691 (42.8)
High	271 (34.0)	174 (21.3)	445 (27.5)

*ANC* antenatal care

^a^Proportions expressed out of those who reported attending ANC for at least once

Table 2 shows the distribution of selected newborn care practices in Luuka and Buyende health sub-districts. Overall, adoption of all beneficial newborn care practices was 11.7 % (189 of 1,616); significantly lower in Luuka (9.4 %, 75 of 797) than in Buyende health sub-district (13.9 %, 114 of 819; Odds Ratio [OR] = 0.64, 95 % Confidence Interval (95 % CI): 0.46, 0.87). Good cord care (83.6 % in Luuka; 95 % in Buyende health sub-district) and immunization of newborn (80.7 % in Luuka health sub-district; 82.5 % in Buyende health sub-district) were the most prevalent newborn care practices reported among mothers. Weighing the baby after birth was fairly practiced in both Luuka (68 %) and Buyende health sub-district (59.8 %). The proportion of mothers reporting good optimal thermal care was lower in both Luuka (40.3 %) and Buyende health sub-district (38.6 %) as was the proportion of mothers reporting good feeding practice (47.2 % in Luuka; 50.6 % in Buyende health sub-district).

**Table 2:** Newborn care practices among mothers resident in Luuka and Buyende health sub-district

Newborn care practices (Yes)	Health sub-district		
	Luuka (*N* = 797)	Buyende (*N* = 819)	Total (*N* = 1,616)
	N (%)	N (%)	N (%)
All Beneficial Newborn Care Practices	75 (9.4)	114 (13.9)	189 (11.7)
Good cord care practice	666 (83.6)	778 (95.0)	1,444 (89.4)
Baby weighed after birth	542 (68.0)	490 (59.8)	1,032 (63.9)
Optimal thermal care	321 (40.3)	316 (38.6)	637 (39.4)
Immunization of newborn	643 (80.7)	676 (82.5)	1,319 (81.6)
Good feeding practice	376 (47.2)	414 (50.6)	790 (48.9)

Table 3 shows bivariate analysis of maternal characteristics and adoption of beneficial newborn care practices. At the bivariate analysis, having primary education for her husband (*p* = 0.002), being a laborer or peasant (*p* = 0.028) and having a first ANC visit in the second trimester (*p* = 0.047) and residing in Buyende health sub-district district (*p* = 0.005), were negatively associated with adoption of all beneficial newborn care practices. On the other hand, Secondary or higher education for the mother (*p* = 0.03), making 3-4 (*p* < 0.001^*^) or 5+ ANC visits’ (*p* = 0.003), delivering under skilled birth attendants (*p* < 0.001^**^), delivery mode (*p* = 0.036), belonging to middle (*p* = 0.002) or higher (*p* = 0.002) socio-economic status, were significantly positively associated with reporting adoption of all beneficial newborn care practices. Mother’s age category, marital status, having first ANC visit at trimester 3, parity, delivery mode, and distance to the health facility where the mother delivered from were not significantly associated with reporting adoption of all beneficial newborn care practices.

**Table 3:** Crude, adjusted odds ratios and 95 % CI associated with adoption of beneficial newborn care practices among mothers in Luuka and Buyende health sub-district, 2011

Maternal characteristics		Beneficial newborn care (*n* = 189)				
		*N* = 1,616			*N* = 1573	
	N	n (%)	Crude OR (95 % CI)	*P*-value	Adjusted OR (95 % CI)	*P*-Value
Age group (*N* = 1512)				0.442		
≤ 24	567	64 (11.3)	1.00			
25–34	622	74 (11.9)	0.83 (0.59, 1.16)	0.272		
≥ 35	323	42 (13.0)	0.80 (0.54, 1.19)	0.274		
Marital Status				0.112		
Not married	144	11 (7.6)	1.00			
Married	1,472	178 (12.1)	1.66 (0.89, 3.13)	0.112		
Education				0.054		
No education	194	27 (15.5)	1.00		1.00	
Primary	1,096	136 (70.9)	1.14 (0.73, 1.78)	0.56	0.83 (0.51, 1.35)	0.457
Secondary or higher	326	26 (13.6)	1.86 (1.05, 3.28)	0.03	0.98 (0.52, 1.86)	0.973
Occupation				0.089		
Salaried or business	113	6 (5.3)	1.00		1.00	
Laborer or peasant farmer	1,357	166 (12.2)	0.40 (0.18, 0.91)	0.028	0.54 (0.23, 1.28)	0.163
House wife or others	146	17 (11.6)	0.43 (0.17, 1.10)	0.076	0.57 (0.21, 1.58)	0.282
Husband’s Education Status				0.002		
No education	292	27 (9.2)	1.00		1.00	
Primary	782	114 (14.6)	0.60 (0.39, 0.93)	0.021	0.63 (0.39, 0.99)	0.045
Secondary or higher	542	48 (8.9)	1.05 (0.64, 1.71)	0.851	0.89 (0.53, 1.50)	0.652
Attended ANC				0.241		
No	33	6 (18.2)	1.00			
Yes	1,583	183 (11.6)	1.70 (0.71, 4.07)	0.241		
Number of ANC Attendances (*n* = 1,583)^a^				0.001^**^		
1-2	303	55 (18.1)	1.00		1.00	
3-4	979	99 (10.1)	2.00 (1.41, 2.86)	0.001^*^	1.69(1.13, 2.52)	0.010
5+	301	29 (9.6)	2.03 (1.26, 3.28)	0.003	1.47(0.86, 2.54)	0.159
Trimester at first ANC Visit (*n* = 1,573)^a^				0.076		
Trimester 1	564	52 (9.2)	1.00		1.00	
Trimester 2	889	112 (12.6)	0.70 (0.49, 0.99)	0.047	0.82 (0.57, 1.20)	0.311
Trimester 3	130	19 (14.6)	0.59 (0.33, 1.04)	0.067	1.07 (0.56, 2.03)	0.837
Parity				0.761		
1	232	24 (10.3)	1.00			
2–4	658	80 (12.2)	0.83 (0.52, 1.35)	0.459		
5+	726	85 (11.7)	0.87 (0.54, 1.40)	0.569		
Delivery Mode				0.036^c^		
Assisted	47	1 (2.1)	1.00			
Normal delivery	1,569	188 (12.0)	6.26 (1.10, 35.55)	0.036^c^	4.83 (0.65,35.78)	0.123
Skilled Delivery				0.001^**^		
No	433	93 (21.5)	1.00		1.00	
Yes	1,183	96 (8.1)	3.10 (2.27, 4.22)	0.001^**^	2.66 (1.92, 3.69)	0.001^**^
Distance to Facility (*N* = 462)^b^				0.157		
> 5 km	232	14 (6.0)	1.00			
< =5 km	230	22 (9.6)	0.61 (0.31, 1.21)	0.157		
Socio-economic Status				0.001^*^		
Low	480	78 (16.2)	1.00		1.00	
Middle	691	69 (10.0)	1.75 (1.24, 2.47)	0.002	1.57 (1.09, 2.26)	0.015
High	445	42 (9.4)	1.86 (1.25, 2.78)	0.002	1.39 (0.90, 2.14)	0.131
Health Sub-District				0.005		
Luuka	797	75 (9.4)	1.00		1.00	
Buyende	819	114 (13.9)	0.64 (0.47, 0.87)	0.005	0.71 (0.51, 0.99)	0.042

^a^Percentages are expressed out of those who attended antenatal care at their last pregnancy

^b^Only 462 (28.6 %) of 1,616 women knew the distance to the health facility where they delivered from. We have included these women only in the bivariate analysis

^c^The *p*-value is based on Fisher’s exact chi2 test; 95 % CI are test based

The Odds ratio (Crude and Adjusted), and the *p*-values are based on the likelihood ratio test

^*^Statistically significant (*p* < 0.001)

^**^highly statistically significant (*p* < 0.0001)

Additionally, Table 3 shows unadjusted and adjusted OR and 95 % CI associated with adoption of all beneficial newborn care practices among mothers who reported at least one ANC visit at their last pregnancy. Results show that higher ANC visits (3-4 vs. 1-2: Adj. OR = 1.69, 95%CI = 1.13, 2.52); skilled delivery (Adj. OR = 2.66, 95%CI = 1.92, 3.69); middle-level socio-economic status (middle vs. low: Adj. OR = 1.57, 95 % CI = 1.09, 2.26) were positively significantly associated with adoption of all beneficial newborn care practices. Residence in Buyende health sub-district (Adj. OR = 0.71, 95 % CI: 0.51, 0.99) was significantly associated with poor adoption of all beneficial newborn care practices.

## Discussion

Our study of adoption of beneficial newborn care practices among mothers in Luuka and Buyende health sub-districts found that only 12 % of mothers in both health sub-districts adopted all beneficial newborn care practices. The low adoption of all beneficial newborn care practices may be related to poor quality of services especially at ANC [41–44] and deep-rooted cultural practices and rituals for newborns [3, 45, 46]. These findings suggest a need for improved sensitization of mothers about the benefits of adopting all beneficial newborn care practices when they come for ANC and the need for interventions that address deep-rooted cultural practices and rituals that still inhibit adoption of newborn care practices [17, 47].

We found that adoption of beneficial newborn practices was associated with skilled delivery and number of ANC attendances, with those attending ANC for 3-4 times more likely to report adoption of all beneficial newborn care practices than those who attended once or twice. These findings suggest that attending ANC for three or four times coupled with increased use of skilled delivery can improve adoption of beneficial newborn care practices [6, 18]. However, these findings should be interpreted in light of the fact that despite nearly three-quarters of mothers delivering under skilled attendance and 62 % of mothers attending ANC services for 3-4 times, only a small proportion of mothers reported adoption of all beneficial newborn care practices. This could partly be due to the fact that the health workers themselves who should have educated mothers about the importance of adoption of beneficial newborn care practices have limited knowledge of these practices [20, 23, 48] and partly due to the continued existence of harmful newborn care practices such as bathing the baby immediately after birth [49], delayed initiation of breastfeeding [50] and putting powder, salty water or lizard droppings on the umbilical cord [3]. Evidence suggests that these practices continue to be practiced even among mothers who have attended ANC and/or delivered under skilled attendance. Note: We acknowledge that some beneficial practices such as optimal thermal care, weighing the baby, and immunization are likely to be done by health facility staff and are dependant of a facility to provide them.

Prior studies in rural Uganda have found that delayed bathing of the baby and putting nothing on the umbilical cord are not acceptable practices among mothers and health care providers [20, 48]. In India, Shah & Dwivedi [49] found that uptake of beneficial newborn care practices is largely hampered by cultural practices, including one known as “*Chatti Puja*” – that restricts wrapping of the baby in clothes or exposing them to sunlight until the seventh day after birth [49]. In Afghanistan, Newbrander et al. [50] found that initiation of breastfeeding may be delayed until up to 3 days after birth – or until a woman has had her first bath after delivery [50]. These findings suggest a need for innovative interventions (e.g. use of mentor-mothers) to address barriers that inhibit adoption of beneficial newborn practices among mothers on the one hand and interventions that target health workers (e.g. through on-the-job, hands-on training) on the other hand to increase their knowledge about the importance of beneficial newborn care practices in improving child survival.

We found that mothers of middle socio-economic status were more likely to practice beneficial newborn care practices compared to those of low socio-economic status. This is in contrast with an earlier study in the same region that reported no significant difference in the adoption of newborn care practices by women’s socio-economic status [1]. Women of higher socio-economic status may be more likely to practice beneficial newborn cares practices because: i) they can easily access health interventions at fixed health facilities which tend to be inequitably distributed, ii) they are able to access skilled delivery, and iii) they can make three or four ANC visits compared to women of low socio-economic status [51, 52]. The mixed findings we see among women of middle socio-economic status compared to low socio-economic status and high compared to low socio-economic status could be because women of high socio-economic status are prone to work pressures, unfavorable work conditions and socio-cultural factors such as maternal and significant other’s beliefs about uptake of recommended newborn care practices such as exclusive breastfeeding [53–56], thus making them less likely to adopt beneficial newborn care practices. Furthermore, the effects of urbanization, maternal education, and socioeconomic status act through the intervening variables of sociocultural factors, health services, employment status of women, availability of breast milk substitutes and use of powder with a resultant effect of failure to adopt beneficial newborn care practices [57]. Waiswa et al. [3] have described this phenomenon among women of high socio-economic status as “modernistic”. On the other hand, the deep-rooted cultural beliefs and practices among women of low socio-economic status are because of lack of relevant health information and access to quality health care. These cultural practices may not be beneficial to newborns [58, 59]. These findings suggest a need for targeting women of low socio-economic class with correct information on the importance of beneficial newborn care practices in influencing a baby’s growth and survival through outreaches or intensified use of community health workers on one hand, and promoting newborn care practices among women of high socio-economic status through quality ANC services at fixed health facilities (both private and public) they attend, improved conditions at work for breastfeeding and supportive policies at national level for uptake of beneficial newborn care practices.

Our study has several limitations. For instance, there is a likelihood that women interviewed for our study did not remember all the beneficial newborn care practices that they practiced when they gave birth to their babies – given that we interviewed mothers who delivered in the past 12 months. It is likely that mothers who delivered exactly 12 months from the time of interview might be less likely to recall all the newborn care practices that they used compared to those who had delivered in a month or so to the time of interview. However, since we did not collect data on the duration between delivery and time of interview, we are unable to assess the extent to which recall bias varied over time. Nevertheless, since women tend to remember what happens to their babies [60–62], it is unlikely that recall bias affected the results reported in a substantial way.

We found that distance to the facility was not significantly associated with adoption of beneficial newborn care practices at the bivariate analysis level. This finding should be interpreted with caution given that 71.4 % of the mothers did not know the distance to the health facility where they delivered from. Indeed, the association between distance to the facility and adoption of beneficial newborn care practices was assessed in only those mothers who knew the distance to the facility. Studies have shown that women reporting long distances to the health facility are less likely to complete the minimum recommended number of antenatal care visits [63–65]. These findings suggest that distance to the facility is an important covariate of the adoption of beneficial newborn care practices among mothers. However, since only 28.6 % of mothers knew the distance to the facility, our study is not able to draw any conclusions about the effect of distance on adoption of beneficial newborn care practices; warranting further inquiry on this aspect.

It is also important to note that the proportions of beneficial newborn care practices reported in this paper are based on self-reports of what women did when they gave birth to their babies. Since we could not verify individual newborn care practices, there is a possibility that some practices may not have been practiced as reported. However, given that almost similar proportions of use of newborn care practices were reported in two separate districts, it is very likely that women’s reports of beneficial newborn care practices might reflect the actual use of these practices in the communities. Additionally, there could be residual confounding, which we were unable to assess, explaining the associations we see in the data.

Despite these limitations, our findings are crucial in the implementation of interventions aimed at improving child growth and survival. The Ministry of Health recommends that pregnant women attend ANC up to four times, and that all mothers deliver under the hands of skilled attendants. At the moment, only 57 % of mothers deliver at the hands of skilled attendants, and ANC attendance declines substantially from 95 % at the first visit to 48 % at the 4^th^ visit [38]. Our findings suggest a need for provision of quality antenatal care, promotion of ANC attendance up to the 4^th^ visit, quality skilled delivery services and skilled delivery attendance in order to increase the proportion of mothers who can and are able to adopt beneficial newborn care practices for the benefit of their babies’ growth and survival.

## Conclusion

Our study shows that adoption of all beneficial newborn care practices was sub-optimal. The independent factors associated with adoption of beneficial newborn care practices were skilled delivery attendance, middle-level socio-economic status and attending ANC for 3-4 visits. These findings suggest a need for improvement of quality of ANC, skilled delivery attendance and the need to target women of both low and high socio-economic status with newborn care health educational messages.

## Abbreviations

ANC: antenatal care
DSS: demography surveillance site
ENC: early newborn care
LIC: low income countries
MANEST: maternal newborn study
OPV: oral polio vaccine
PCA: principle component analysis
SES: socio-economic status
UNICEF: United Nations Children’s Fund
VHT: village health team
WHO: World Health Organization
